# P-1448. Barriers and Facilitators of Respiratory Syncytial Virus (RSV) Vaccine Confidence Among a Community-Based Sample of Latino Older Adults in San Francisco

**DOI:** 10.1093/ofid/ofaf695.1634

**Published:** 2026-01-11

**Authors:** Fernanda Amaya, Lisa Geronimo, Sara Colom Brana, Noel Sergio Leon, Shalom Bandi, Francisco J Herrera, Douglas Black, Dave Graham-Squire, John Sauceda, Susana Rojas, Julia Lechuga, Diane Havlir, Carina Marquez

**Affiliations:** University of California San Francisco, Colma, California; University of California San Francisco, Colma, California; UCSF, San Francisco, California; UCSF, San Francisco, California; Latino Task Force, San Francisco, California; Nuevo Sol / Latino Task Force, San Francisco, California; University of California, San Francisco, San Francisco, California; UCSF, San Francisco, California; University of California San Francisco, Colma, California; Unidos en Salud, San Francisco, California; The University of California San Francisco, San Francisco, California; UCSF, San Francisco, California; University of California, San Francisco, San Francisco, California

## Abstract

**Background:**

The new and effective RSV vaccine can reduce morbidity from RSV among older adults, yet uptake is below goal and there are differences in uptake across race/ethnicity and income groups. Thus, we sought to describe barriers and facilitators to RSV vaccine uptake among Latino older adults and likelihood of recommending the vaccine to their family members.

**Figure:**
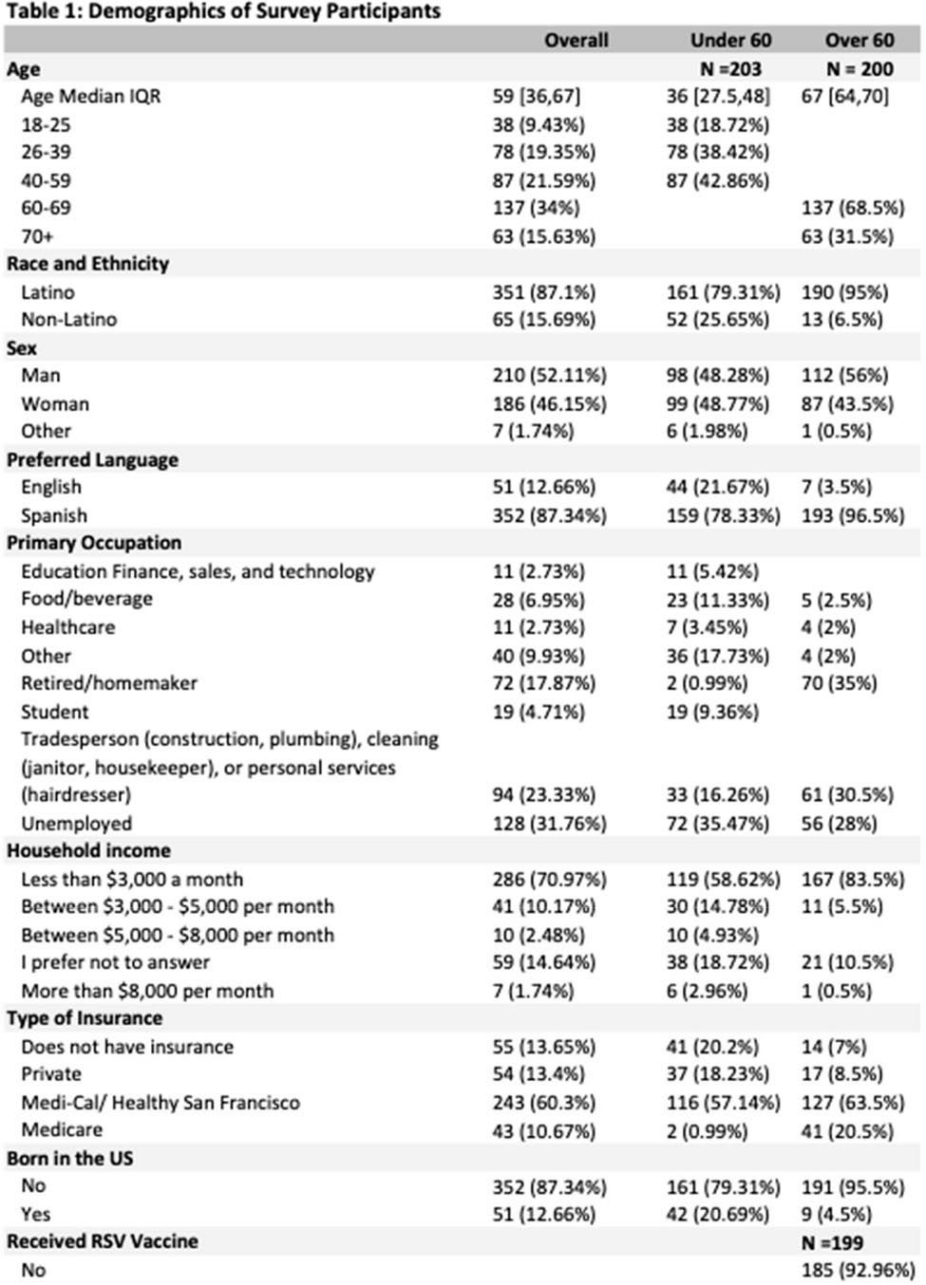
Demographics of Survey Participants

**Figure:**
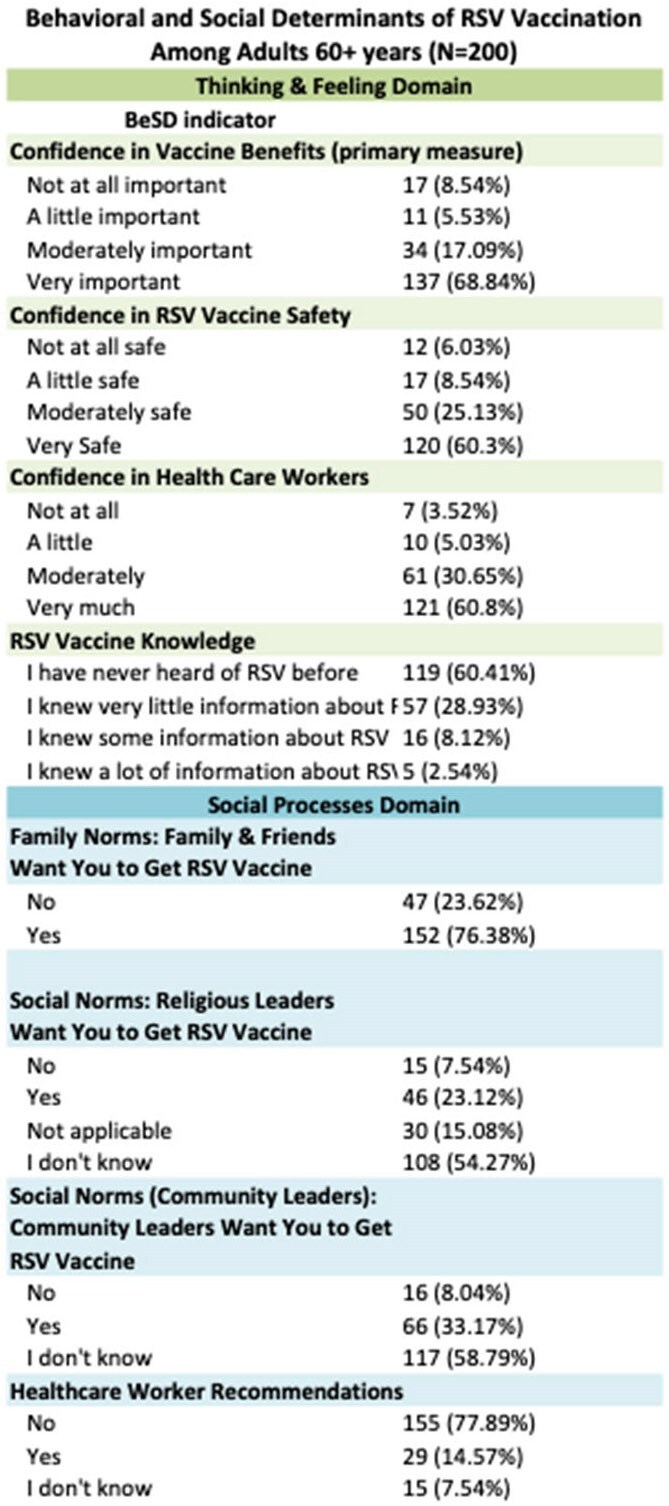
Behavioral and Social Determinants of RSV Vaccination Among adults 60+ years

**Methods:**

In this cross-sectional study, we administered surveys to a convenience sample of adults at Latino-focused community-based organizations and churches. Surveys assessed barriers and facilitators of RSV vaccination using Behavioral and Social Drivers (BeSD) conceptual model (Figure 1). We assessed sociodemographic variables and the odds of being motivated to get the RSV vaccine (60+ years) with a logistic regression, adjusting by age, sex, and race.

**Figure:**
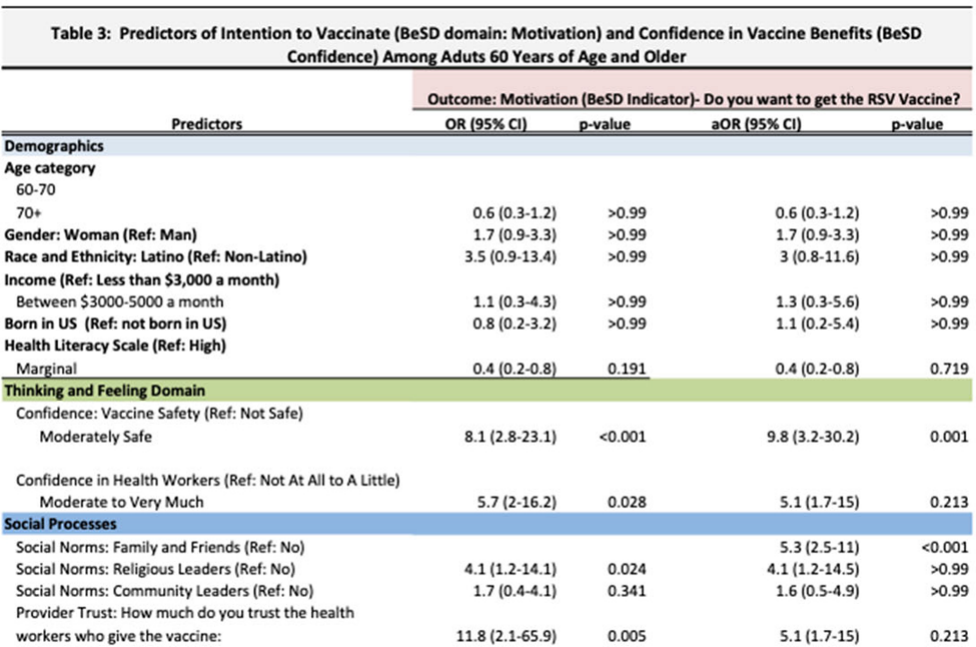
Predictors of Intention to Vaccinate (BeSD Domain: Motivation) and Confidence in Vaccine Benefits (BeSD Confidence) Among Adults 60 Years of Age and Older

**Figure:**
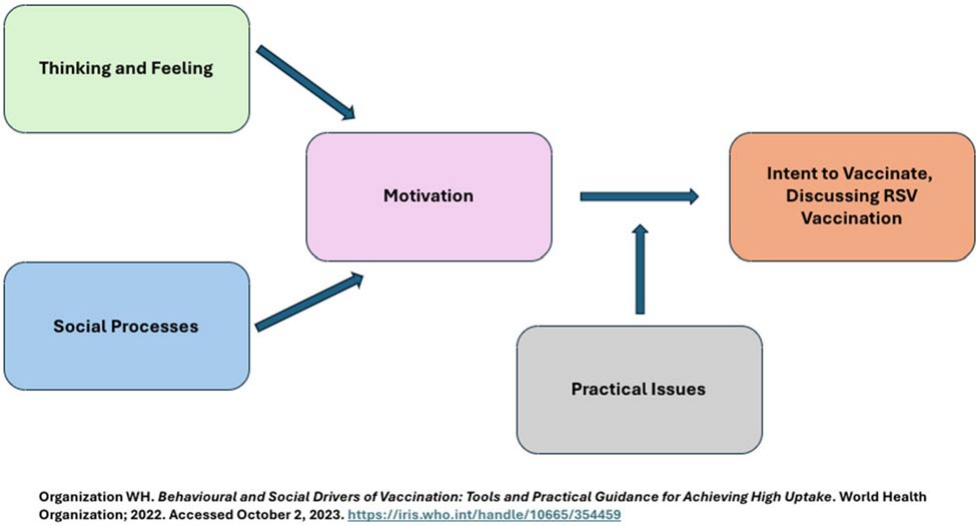
Behavioral and Social Drivers of Vaccination

**Results:**

From November 2024 to February 2025, we administered 403 surveys (200 among adults 60+). Majority of participants were Latino (87%) and born outside of the US (87%) (Table 1). Among adults 60+, 3% received the RSV vaccine, whereas 47% received the zoster vaccine and 82% received a COVID booster. Prior to the survey, 58% had never heard of the RSV virus. Once made aware of RSV and vaccine eligibility, 67% were motivated to get vaccinated and 68% felt the vaccine was very important (Table 2). Confidence in vaccine benefits was highly correlated with motivation, with 71% of people who felt the vaccine was important expressing strong motivation to get the vaccine. After adjusting for age, sex, and race, older adults’ motivation to get the RSV vaccine was positively associated with confidence in vaccine safety (aOR: 9.8, 95%CI: 3.2-30.2), trust in health workers (aOR: 5.1, 95%CI: 1.7-15), and social norms-- family and friends want them to get vaccine (aOR: 5.3, 95%CI: 2.5-11) (Table 3). Practical challenges were reported, with 73% expressing cost concerns. Among people 18-59 years, 77% discuss health matters with their older family members and 70% would recommend the RSV vaccine.

**Conclusion:**

After hearing about RSV vaccine eligibility, motivation to get vaccinated was high, though actual vaccine uptake was only 3%. Strategies to increase uptake should include education from trusted sources including health care providers, family members and friends about vaccine eligibility and access.

**Disclosures:**

Diane Havlir, MD, VIiV: Grant/Research Support

